# Supplementary material for: Fucosyltransferase 2 inhibitors: Identification via docking and STD-NMR studies
Source: PLoS One. 2021 Oct 14;16(10):e0257623. doi: 10.1371/journal.pone.0257623 (PMC8516197; doi:10.1371/journal.pone.0257623)
Supplement: S1 Fig — Red color indicates the ligand RMSD, while blue is for protein. During the total run time of simulation, the acceptor and donor were stable in their respective pockets. (DOCX) [file pone.0257623.s001.docx]

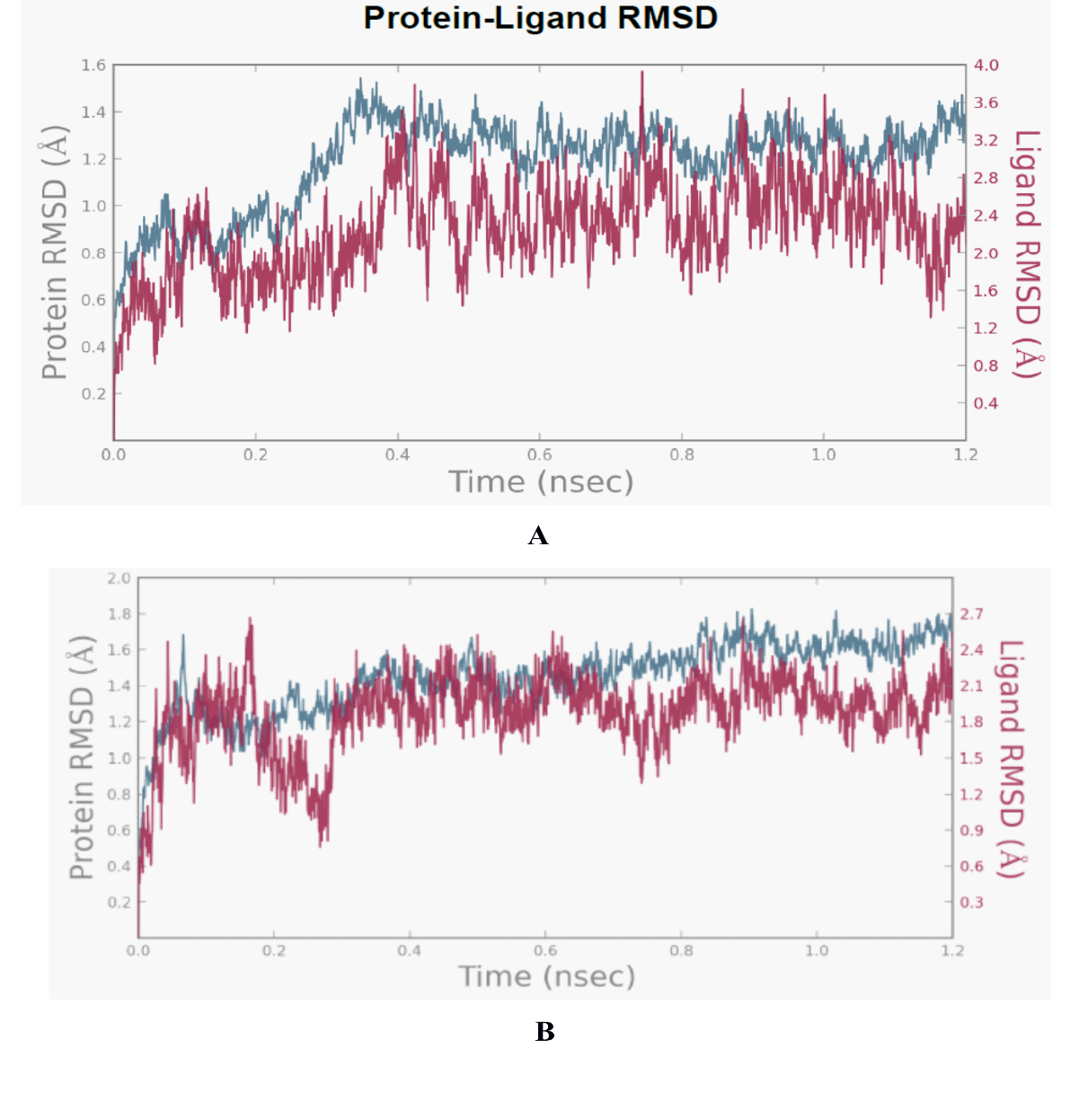


**Figure-S1: Protein ligand RMSD:** Simulation of the part of the A. donor (3ZY5) and B. acceptor (1W3F). Red color indicates the ligand RMSD, while blue is for protein. During the total run time of simulation, the acceptor and donor were stable in their respective pockets.
