# Supplementary figures and images for: Fucosyltransferase 2 inhibitors: Identification via docking and STD-NMR studies
Source: PLoS One. 2021 Oct 14;16(10):e0257623. doi: 10.1371/journal.pone.0257623 (PMC8516197; doi:10.1371/journal.pone.0257623)

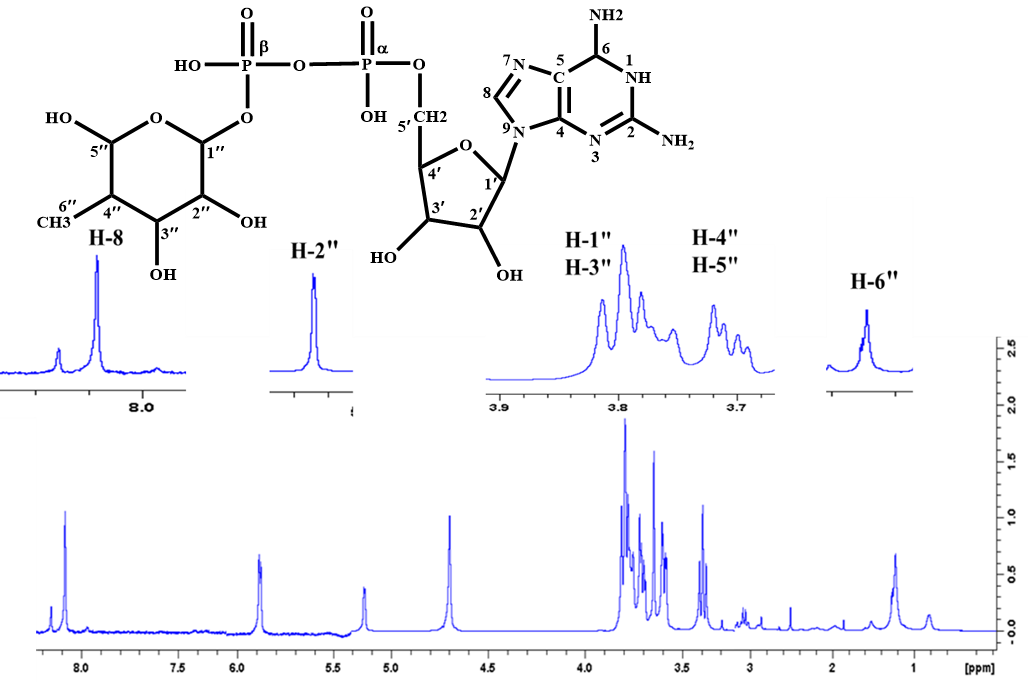


**Figure-S2:** 1H-NMR of receptor-ligand complex. Resonances of ligand (GDP-Fucose) are presented.

Supplement: S2 Fig — Resonances of ligand (GDP-Fucose) are presented. (DOCX) [file pone.0257623.s002.docx]

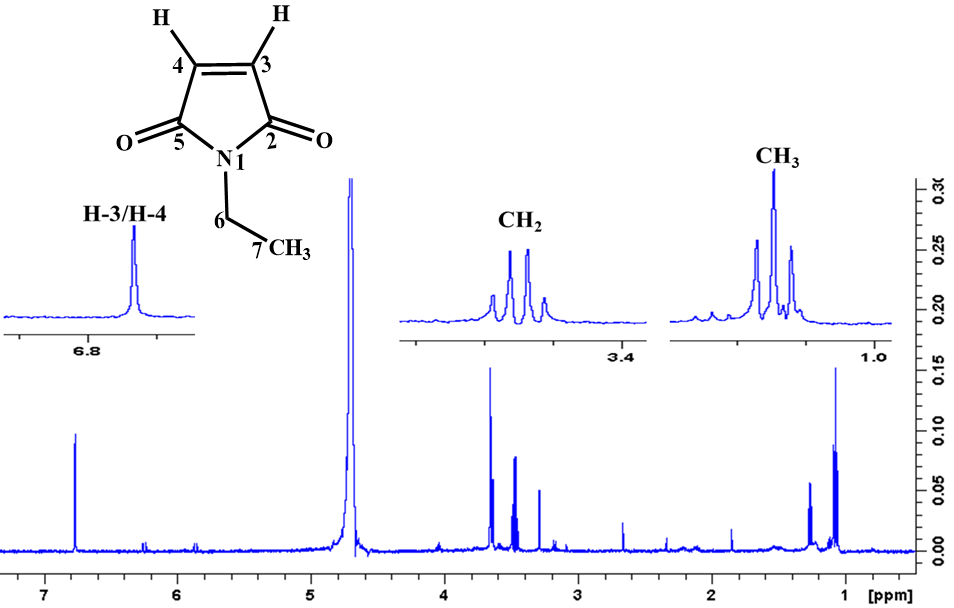


**Figure-S3:** 1H-NMR of receptor-ligand complex. Resonances of ligand (*N*-Ethylmaleimide) are presented.

Supplement: S3 Fig — Resonances of ligand (N-Ethylmaleimide) are presented. (DOCX) [file pone.0257623.s003.docx]

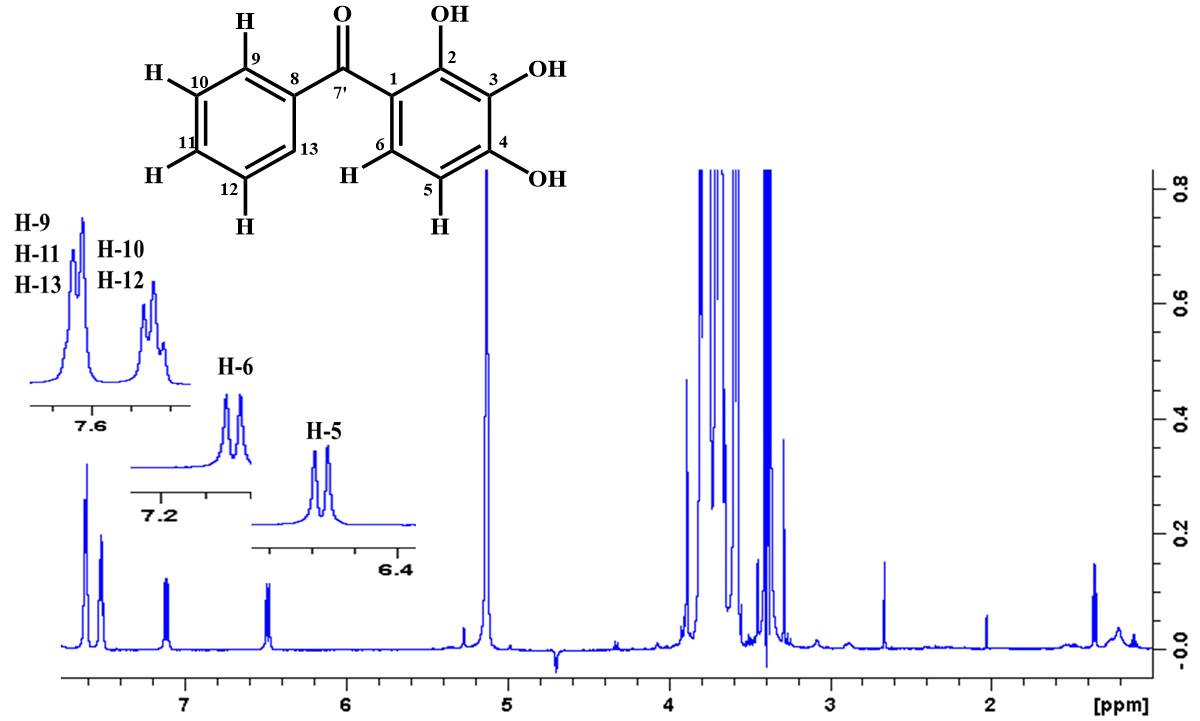


**Figure-S4:** 1H-NMR of receptor-ligand complex. Resonances of ligand (ligand **1**) are presented.

Supplement: S4 Fig — Resonances of ligand (ligand 1) are presented. (DOCX) [file pone.0257623.s004.docx]

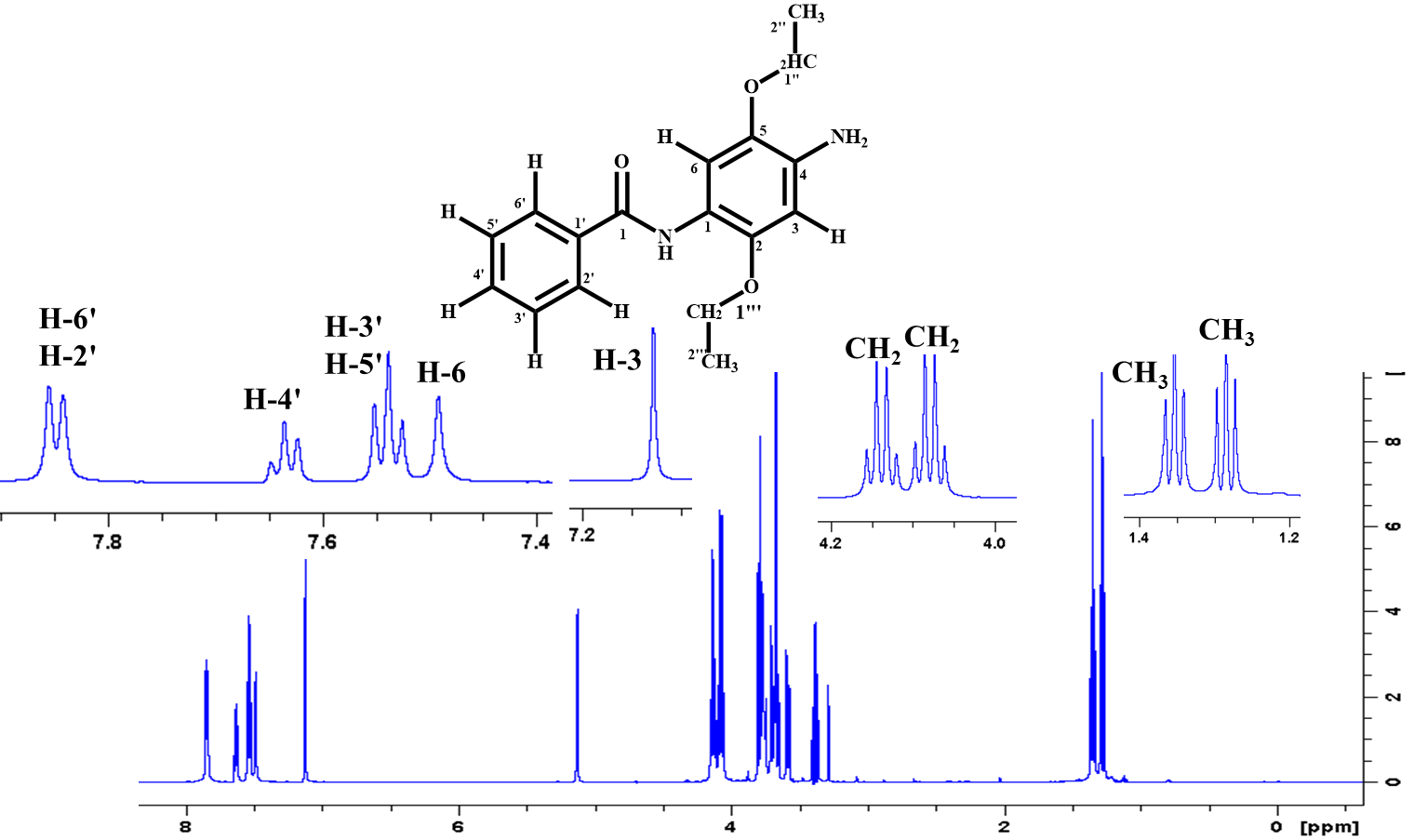


**Figure-S5:** 1H-NMR of receptor-ligand complex. Resonances of ligand (ligand **2**) are presented.

Supplement: S5 Fig — Resonances of ligand (ligand 2) are presented. (DOCX) [file pone.0257623.s005.docx]

**
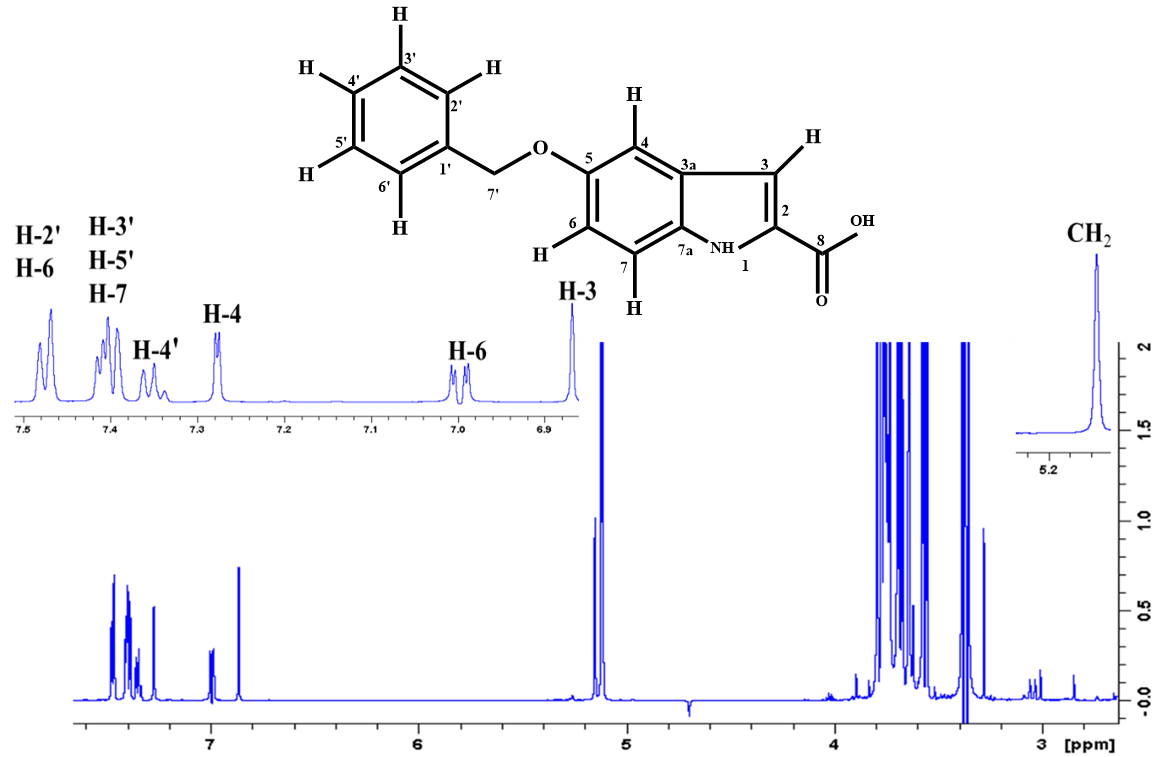
**

**Figure-S6:** 1H-NMR of receptor-ligand complex. Resonances of ligand (ligand **3**) are presented.

Supplement: S6 Fig — Resonances of ligand (ligand 3) are presented. (DOCX) [file pone.0257623.s006.docx]

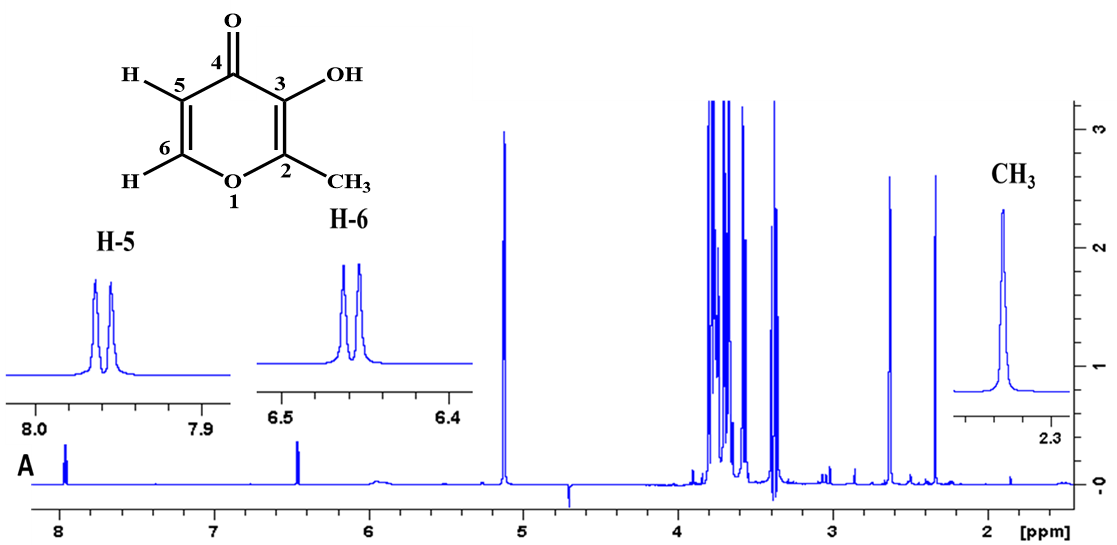


**Figure-S7:** 1H-NMR of receptor-ligand complex. Resonances of ligand (ligand **4**) are presented.

Supplement: S7 Fig — Resonances of ligand (ligand 4) are presented. (DOCX) [file pone.0257623.s007.docx]

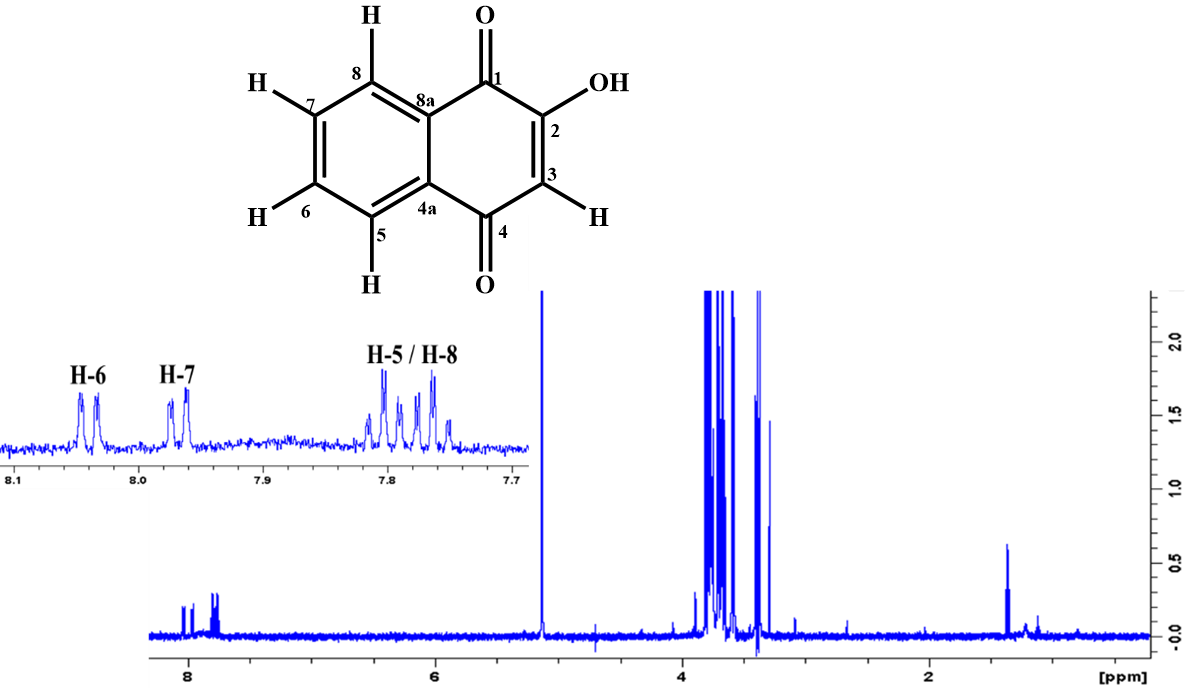


**Figure-S8:** 1H-NMR of receptor-ligand complex. Resonances of ligand (ligand **5**) are presented.

Supplement: S8 Fig — Resonances of ligand (ligand 5) are presented. (DOCX) [file pone.0257623.s008.docx]

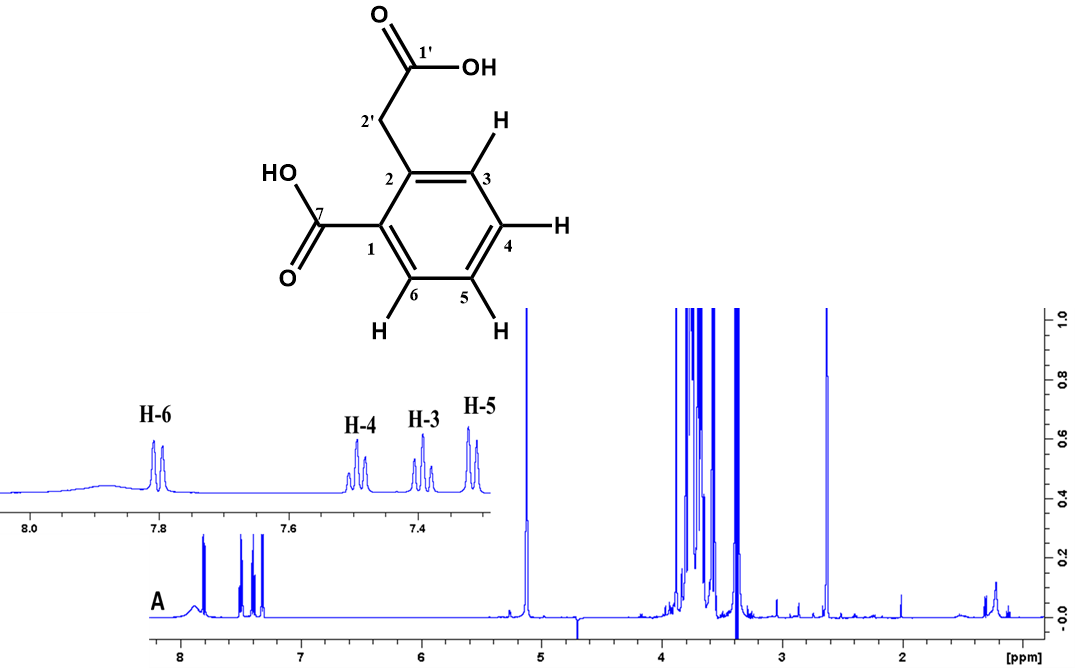


**Figure-S9:** 1H-NMR of receptor-ligand complex. Resonances of ligand (ligand **26**) are presented.

Supplement: S9 Fig — Resonances of ligand (ligand 26) are presented. (DOCX) [file pone.0257623.s009.docx]

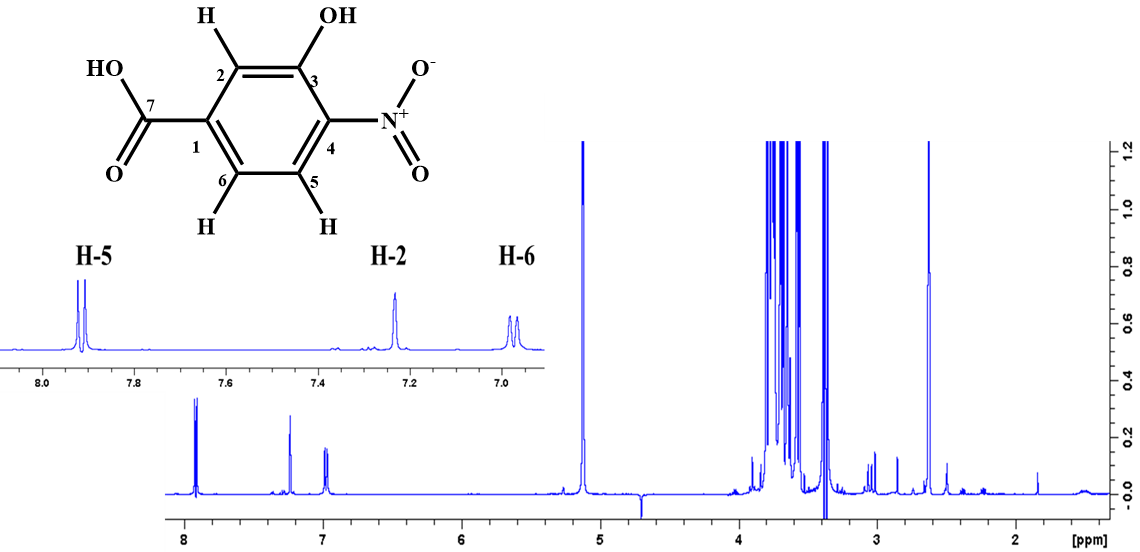


**Figure-S10:** 1H-NMR of receptor-ligand complex. Resonances of ligand (ligand **27**) are presented.

Supplement: S10 Fig — Resonances of ligand (ligand 27) are presented. (DOCX) [file pone.0257623.s010.docx]

**
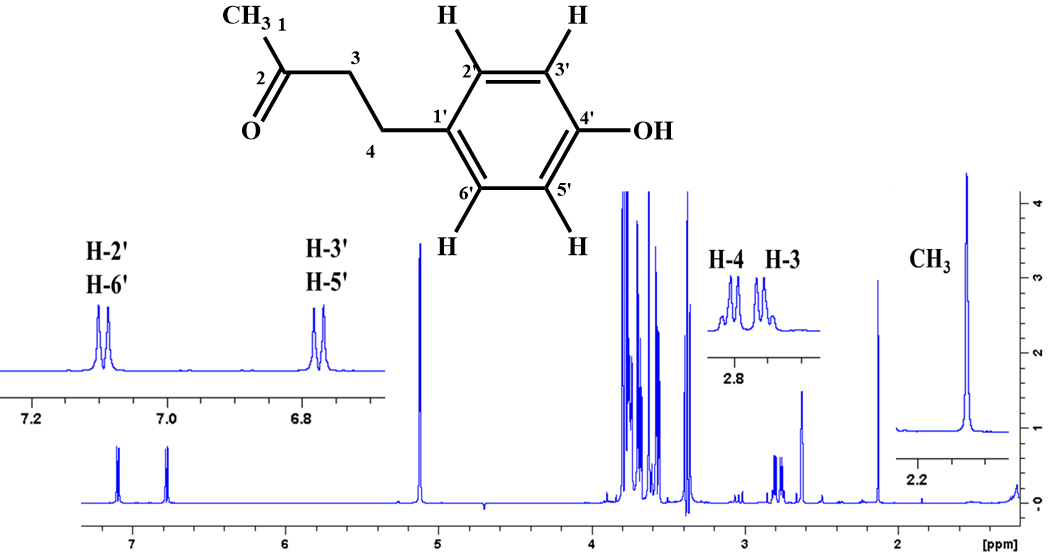
**

**Figure-S11:** 1H-NMR of receptor-ligand complex. Resonances of ligand (ligand **28**) are presented.

Supplement: S11 Fig — Resonances of ligand (ligand 28) are presented. (DOCX) [file pone.0257623.s011.docx]

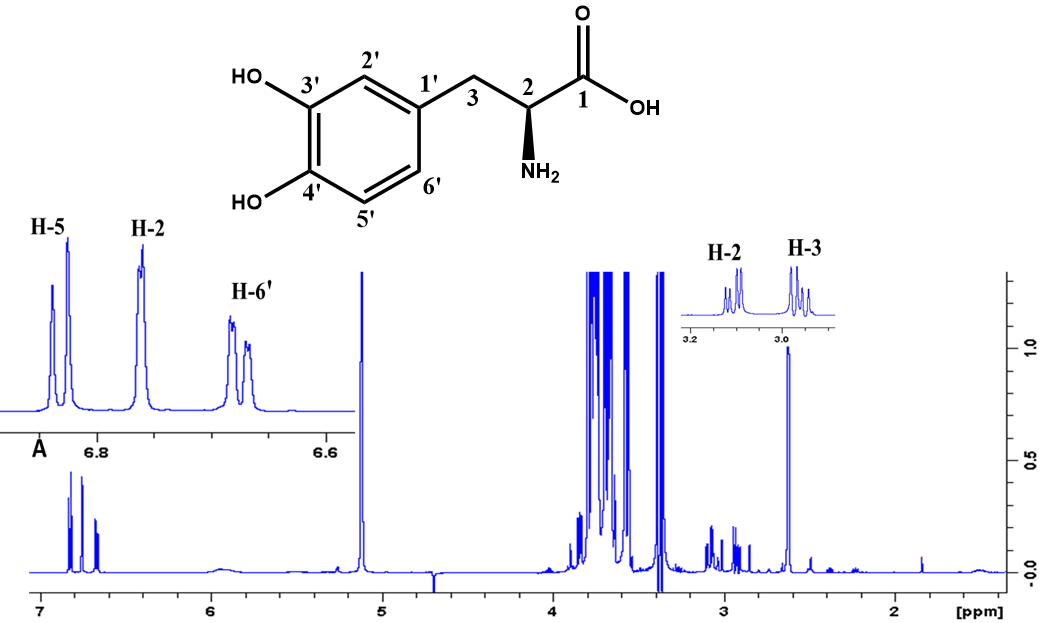


**Figure-S12:** 1H-NMR of receptor-ligand complex. Resonances of ligand (ligand **29**) are presented.

Supplement: S12 Fig — Resonances of ligand (ligand 29) are presented. (DOCX) [file pone.0257623.s012.docx]
