# Supplementary material for: Fucosyltransferase 2 inhibitors: Identification via docking and STD-NMR studies
Source: PLoS One. 2021 Oct 14;16(10):e0257623. doi: 10.1371/journal.pone.0257623 (PMC8516197; doi:10.1371/journal.pone.0257623)
Supplement: S1 Table — (DOCX) [file pone.0257623.s013.docx]

**Table-S1: Docking scores and binding affinity estimation of fragments against donor and acceptor domains of FUT2.**

| **S.No.** | **Fragments Structures** | **Docking Scores** | | **MMGBSA dG Bind (ΔGbind, kcal/mol)** | |
| --- | --- | --- | --- | --- | --- |
|  |  | **3ZY5** | **1W3F** | **3ZY5** | **1W3F** |
| 1 |   Phenyl-(2,3,4-trihydroxyphenyl)methanone | -9.08 | -8.54 | -94.92 | -30 |
| 2 |   *N*-(4-Amino-5-ethoxy-2-(methoxymethyl)phenyl)benzamid | -8.21 | -7.41 | -74.13 | -51 |
| 3 |   5-Phenethyl-1*H*-indole-2-carboxylic acid | -7.46 | -6.16 | -96.77 | -71 |
| 4 |   3-Hydroxy-2-methyl-4*H*-pyran-4-one | -5.55 | -5.25 | -92.98 | -.31 |
| 5 |   2-Hydroxynaphthalene-1,4-dione | -5.25s | -5.07 | -60.17 | -10 |
| 6 |   5-Bromo-3-hydroxyindolin-2-one | -4.62 | -2.81 | -42.31 | -24 |
| 7 |   6-Aminopyridine-2,4-diol | -3.92 | -2.76 | -71.57 | -21 |
| 8 |   3-Bromo-5-chloro-2-hydroxybenzaldehyde | -7.26 | -2.72 | -58.35 | -23 |
| 9 |   Quinolin-8-ol | -3.76 | -2.64 | -83.33 | -24 |
| 10 |   3-Bromo-5-chloro-2 hydroxybenzaldehyde | -4.54 | -2.64 | -75.28 | -12 |
| 11 |   3-Bromo-5-chloro-2-hydroxybenzaldehyde | -4.32 | -2.34 | -101.3 | -29 |
| 12 |   2-Amino-4-chlorophenol | -4.76 | -2.30 | -53.73 | -33 |
| 13 |   2-Amino-4-methylphenol | -4.52 | -2.29 | -63.35 | -73 |
| 14 |   1-(*p*-Tolyl) urea | -5.23 | -2.29 | -81.16 | -19 |
| 15 |   Benzyl-(2,5-dioxopyrrolidin-1-yl)-carbonate | -4.93 | -2.24 | -77.73 | -20 |
| 16 |   Benzene-1,3,5-triol | -4.97 | -2.27 | -58.18 | -22 |
| 17 |   1*H*-Benzo[d]-imidazole-2-thiol | -4.80 | -2.1 | -61.75 | -18 |
| 18 |   Phenyl-(pyridin-2-yl)-methanone | -5.01 | -5.01 | -94.09 | -46 |
| 19 |   Benzyl carbamate | -4.42 | -1.97 | -51.35 | -19 |
| 20 |   Anthracene-9,10-dione | -3.96 | -1.92 | -70.4 | -36 |
| 21 |   Isophthalaldehyde | -3.86 | -1.77 | -58.66 | -13 |
| 22 |   2-Hydroxy-1,2-diphenylethan-1-one | -3.32 | -1.75 | -61.75 | -12 |
| 23 |   2-Thioxo-2,3-dihydropyrimidin-4(1*H*)-one | -2.876 | -1.74 | -87.04 | -20 |
| 24 |   1-(2,5-Dioxoimidazolidin-4-yl)-urea | -3.64 | -1.75 | -46.57 | -23 |
| 25 |   5-Amino-1*H*-imidazole-4-carboxamide | -4.62 | -2.81 | -72.65 | -31 |
| 26 |   2-(Carboxymethyl) benzoic acid | -7.13 | -9.47 | -59.6 | -44.56 |
| 27 |   3-Hydroxy-4-nitrobenzoic acid | -6.54 | -7.09 | -88.86 | -49.79 |
| 28 |   4-(4-Hydroxyphenyl) butan-2-one | -6.12 | -6.55 | -55.19 | -45.23 |
| 29 | **  *(S)-*2-Amino-3-(3,4-dihydroxyphenyl) propanoic acid | -5.46 | -6.16 | -40.01 | -21.91 |
| 30 |   2-Methoxybenzoic acid | -5.41 | -3.13 | -68.17 | -28.97 |
| 31 |   (3-(Trifluoromethyl) phenyl) boronic acid | -5.4 | -5.07 | -70.65 | -37.57 |
| 32 |   3-Hydroxy-2-naphthoic acid | -4.97 | -3.03 | -70.49 | -22.93 |
| 33 |   7-Mercapto-4-methyl-*2H*-chromen-2-one | -6.33 | -3.00 | -46.24 | -17.9 |
| 34 |   6-Aminopyrimidine-2*(1H)-*thione | -4.97 | -2.98 | -31.16 | -28.8 |
| 35 |   4-(Hydroxymethyl)-2-methoxyphenol | -4.04 | -2.96 | -54.39 | -13.59 |
| 36 |   5-Chloro-2-hydroxybenzoic acid | -5.42 | -2.92 | -60.7 | -21.78 |
| 37 |   2,4,6-Trihydroxybenzoic acid | -4.75 | -2.88 | -39.97 | -20.42 |
| 38 |   9*H*-Thioxanthen-9-one | -4.62 | -2.81 | -39.45 | -22.73 |
| 39 |   2*H*-Chromene-3-carboxylic acid | -3.92 | -2.76 | -40.52 | -14.26 |
| 40 |   2-Mercaptonicotinic acid | -7.24 | -2.72 | -50.25 | -23.4 |
| 41 |   2,6-Dihydroxyisonicotinic acid | -4.84 | -2.72 | -59.38 | -33.82 |
| 42 |   2-*(p-tolyl)* acetic acid | -3.76 | -2.64 | -77.85 | -27.44 |
| 43 |   2,3-Dihydro-1*H*-inden-5-ol | -4.54 | -2.64 | -59.89 | -19.78 |
| 44 |   5-Hydroxy-2-nitrobenzaldehyde | -4.42 | -2.63 | -48.07 | -25.28 |
| 45 |   Ethyl-2-oxopiperidine-3-carboxylate | -5.30 | -2.63 | -43.34 | -26.64 |
| 46 |   [1,1'-Biphenyl]-2-carboxylic acid | -3.07 | -2.62 | -54.73 | -26.31 |
| 47 |   2,4,6-Tribromoaniline | -3.06 | -2.61 | -39.05 | -27.35 |
| 48 |   Thianthrene | -4.20 | -2.59 | -53.83 | -25.82 |
| 49 |   (4-Methoxyphenyl)-(phenyl)methanone | -4.33 | -2.56. | -59.49 | -37.02 |
| 50 |   5-Chloroquinolin-8-ol | -5.41 | -3.13 | -42.31 | -44.56 |
