# Supplementary material for: Fucosyltransferase 2 inhibitors: Identification via docking and STD-NMR studies
Source: PLoS One. 2021 Oct 14;16(10):e0257623. doi: 10.1371/journal.pone.0257623 (PMC8516197; doi:10.1371/journal.pone.0257623)
Supplement: S2 Table — (DOCX) [file pone.0257623.s014.docx]

**Table-S2: Types of ligand-receptor interactions in donor and acceptor binding sites of receptor protein FUT2.**

| **Binding sites of receptor protein FUT2** | **Amino acid residues of receptor protein FUT2** | **Functional groups of ligand** | **Type of Interaction** |
| --- | --- | --- | --- |
| **Compound 1** | | | |
| Donor binding site of receptor | Arg240, Ser353  Gly42 | OH at C-2'  CO at C-1 | H-bonding  H-bonding |
|  | Phe357 | Phenolic ring | π-π stacking |
| Acceptor binding site of receptor | Asp133  Trp134  Arg123 | OH at C-3, C-4  OH at C-4  OH at C-2 | H-bonding  H-bonding  H-bonding |
| **Compound 2** | | | |
| Donor binding site of receptor | His238 | CO at C-1 | H-bonding |
| Acceptor binding site of receptor | Arg123 | CO at C-1 | H-bonding |
|  |  | Phenolic ring | π-cation |
| **Compound 3** | | | |
| Donor binding site of receptor | Thr356 | CO at C-2 | H-bonding |
|  | Phe357 | Phenolic ring | π-π stacking |
| Acceptor binding site of receptor | Arg76, Trp134 | CO at C-2 | H-bonding |
|  | Asp133 | Indole ring | π-cation interaction |
| **Compound 4** | | | |
| Donor binding site of receptor | Ser355, Arg240  Asn43 | CO at C-4  O at C-1 | H-bonding  H-bonding |
| Acceptor binding site of receptor | Trp126, Arg123  Asn128  Arg76 | CO at C-1  OH at C-1  OH at C-1 | H-bonding  H-bonding  H-bonding |
| **Compound 5** | | | |
| Donor binding site of receptor | Arg40, Asn43  Gly42  Arg240, Ser355 | CO at C-1  CO at C-2  CO at C-4 | H-bonding  H-bonding  H-bonding |
|  | Phe261 | Phenolic ring | π-π stacking |
| Acceptor binding site of receptor | Arg123  Asp133, His141 | CO at C-1  OH at C-2 | H-bonding  H-bonding |
| **Compound 26** | | | |
| Donor binding site of receptor | Asn43 | OH at C-1' | H-bonding |
|  | Arg240 | Phenolic ring | π-cation interaction |
| Acceptor binding site of receptor | Arg76  Asp133  Arg123 | O at C-1'  OH at C-1', C-7  O at C-1' | H-bonding  H-bonding  H-bonding |
|  | Arg123 | Phenolic ring | π-cation interaction |
| **Compound 27** | | | |
| Donor binding site of receptor | Asp334  Phe357, His238 | OH at C-7  NO_2_ at C-4 | H-bonding  H-bonding |
| Acceptor binding site of receptor | Asp133, His141, Arg123 | OH at C-7, C-3  NO_2_ at C-4 | H-bonding  H-bonding, |
|  | Tyr139, Trp126, | NO_2_ at C-4 | π-cation interaction |
| **Compound 28** | | | |
| Donor binding site of receptor | Gly42  Asp334 | CO at C-2  OH at C-4' | H-bonding  H-bonding |
|  | Phe357 | Phenolic ring | π-π stacking interaction |
| Acceptor binding site of receptor | Arg123  Asp133 | CO at C-2  OH at C-4' | H-bonding  H-bonding |
|  | Trp126 | Phenolic ring | π-π stacking interaction |
| **Compound 29** | | | |
| Donor binding site of receptor | - | - | No interaction |
| Acceptor binding site of receptor | - | - | No interaction |
