# Supplementary material for: Fucosyltransferase 2 inhibitors: Identification via docking and STD-NMR studies
Source: PLoS One. 2021 Oct 14;16(10):e0257623. doi: 10.1371/journal.pone.0257623 (PMC8516197; doi:10.1371/journal.pone.0257623)
Supplement: Attachment — Submitted filename: Response to Reviewers.docx [file pone.0257623.s015.docx]

**Response to Reviewer’s Comments**

| **Reviewer: 1** | |
| --- | --- |
| **Comment** | **Answer** |
| **1**- The authors are suggested to concise the introduction part. | **1**- The introduction part has been concised accordingly. |
| **2-** Authors should also provide no. of interactions during docking. | **2**- The detail of number and types of interactions predicted during docking studies are now presented in the supplementary section **Table-S3** |
| **3-** Article needs to improve quality of the figures. | **3**- The quality of figures has now been improved accordingly. |
| **4-** The authors are suggested to give the recent references in support of this work. | **4**- The manuscript has been updated with the recent references. |
| **Reviewer: 2** | |
| I would like to suggest the author to give the clear NMR for all the complexes. | All the clear NMR spectra for the complexes have given in the supporting information figures (S2-S12). |
